# Predictive potential of angiopoietin-2 in a mCRC subpopulation treated with vanucizumab in the McCAVE trial

**DOI:** 10.3389/fonc.2023.1157596

**Published:** 2023-05-03

**Authors:** Cláudia S. Ferreira, Galina Babitzki, Irina Klaman, Oliver Krieter, Katharina Lechner, Johanna Bendell, Suzana Vega Harring, Florian Heil

**Affiliations:** ^1^ Roche Pharma Research and Early Development, Roche Innovation Center Munich, Penzberg, Germany; ^2^ PHCS Biostatistics & Data Management, Roche Innovation Center Munich, Penzberg, Germany; ^3^ Sarah Cannon Research Institute and Tennessee Oncology, Nashville, TN, United States

**Keywords:** angiopoietin-2, predictive biomarkers, VEGF, KRAS mutation status, phase II clinical trial, colorectal cancer, vanucizumab, bevacizumab

## Abstract

**Introduction:**

Angiopoetin-2 (Ang-2) is a key mediator of tumour angiogenesis. When upregulated it is associated with tumour progression and poor prognosis. Anti-vascular endothelial growth factor (VEGF) therapy has been widely used in the treatment of metastatic colorectal cancer (mCRC). The potential benefit of combined inhibition of Ang-2 and VEGF-A in previously untreated patients with mCRC was evaluated in the phase II McCAVE study (NCT02141295), assessing vanucizumab versus bevacizumab (VEGF-A inhibitor), both in combination with mFOLFOX-6 (modified folinic acid [leucovorin], fluorouracil and oxaliplatin) chemotherapy. To date, there are no known predictors of outcome of anti-angiogenic treatment in patients with mCRC. In this exploratory analysis, we investigate potential predictive biomarkers in baseline samples from McCAVE participants.

**Methods:**

Tumour tissue samples underwent immunohistochemistry staining for different biomarkers, including Ang-2. Biomarker densities were scored on the tissue images using dedicated machine learning algorithms. Ang-2 levels were additionally assessed in plasma. Patients were stratified by KRAS mutation status determined using next generation sequencing. Median progression-free survival (PFS) for each treatment group by biomarker and KRAS mutation was estimated using Kaplan–Meier plots. PFS hazard ratios (and 95% confidence intervals) were compared using Cox regression.

**Results:**

Overall low tissue baseline levels of Ang-2 were associated with longer PFS, especially in patients with wild-type *KRAS* status. In addition, our analysis identified a new subgroup of patients with KRAS wild-type mCRC and high levels of Ang-2 in whom vanucizumab/mFOLFOX-6 prolonged PFS significantly (log-rank p=0.01) by ~5.5 months versus bevacizumab/mFOLFOX-6. Similar findings were seen in plasma samples.

**Discussion:**

This analysis demonstrates that additional Ang-2 inhibition provided by vanucizumab shows a greater effect than single VEGF-A inhibition in this subpopulation. These data suggest that Ang-2 may be both a prognostic biomarker in mCRC and a predictive biomarker for vanucizumab in KRAS wild-type mCRC. Thus, this evidence can potentially support the establishment of more tailored treatment approaches for patients with mCRC.

## 1 Introduction

Vascular endothelial growth factor (VEGF) is a key mediator of angiogenesis, a pivotal process in tumour growth and metastasis (1, 2), and a regulator of vascular permeability (3). Regimens based on anti-VEGF agents, such as bevacizumab, have led to improvements in outcomes for some patients with colorectal cancer (CRC) (4–8). However, the efficacy of these agents can be limited by the activation of compensatory alternative angiogenic pathways that provide the tumour with an escape mechanism(s) allowing angiogenesis to continue (9). One suggested option for obtaining further control of angiogenesis would be to combine anti-VEGF agents with other compounds that are directed towards these angiogenic escape pathways and have complementary modes of action (10, 11).

Resistance to VEGF-targeted therapies may be partly mediated by angiopoietin-2 (Ang-2), a Tie2 receptor ligand and a key regulator of angiogenesis (11, 12). Ang-2 is upregulated in several tumour types, including metastatic CRC (mCRC), and is associated with poor prognosis (13–16). Like VEGF, Ang-2 is a driver of vascular destabilisation (17), and high levels have been found to counteract the vascular-normalising effects of anti-VEGF therapy (18). In patients with mCRC receiving bevacizumab-containing therapy, those with elevated serum levels of Ang-2 had worse survival outcomes than patients with low Ang-2 levels (19). These findings suggest that Ang-2 may be a useful biomarker in patients receiving anti-angiogenic/anti-VEGF treatment and may provide a rationale for a treatment strategy involving dual inhibition of both VEGF and Ang-2.

Vanucizumab (RO5520985) is a humanised immunoglobulin (Ig)G-1-like bispecific monoclonal antibody targeting both VEGF-A and Ang-2 that has shown anti-tumour, anti-angiogenic and anti-metastatic effects in preclinical studies (20). In phase I studies, vanucizumab has been associated with marked post-infusion reductions in circulating unbound VEGF-A and Ang-2 in plasma, tumour and wound-healing biopsies, thus confirming its mechanism of action (21). It has also demonstrated an acceptable safety profile and favourable pharmacokinetic/pharmacodynamic effects in patients with advanced cancer (22). In the phase II McCAVE (Vanucizumab plus mFOLFOX-6 Versus Bevacizumab plus mFOLFOX-6 in Patients with Previously Untreated Metastatic Colorectal Carcinoma) study, conducted in previously untreated patients with mCRC, vanucizumab and bevacizumab (both plus modified [m] folinic acid [leucovorin], 5-fluorouracil and oxaliplatin [FOLFOX-6]) showed similar clinical efficacy in terms of progression-free survival (PFS) and overall response rates (23). Hence, the efficacy seen with both agents appeared to be mediated mainly *via* VEGF-blockade. Of note, overall outcomes were worse in both treatment arms in patients with higher than median baseline Ang-2 plasma levels versus those with low/equal Ang-2 levels in the total study population (23).

There is strong evidence that Kirsten rat sarcoma virus oncogene (*KRAS*) mutation status is a predictive biomarker in mCRC in anti-epidermal growth factor receptor (EGFR) therapy (24). The KRAS protein acts as a regulator of downstream signalling pathways, such as cell proliferation and survival, and ultimately tumorigenesis (25). Mutations in this protein therefore promote angiogenesis, and impact the prognosis and treatment of CRC (26). *KRAS* mutations have been reported in up to ~50% of patients with CRC (26, 27) and in 36% of those with mCRC (28). Shorter survival outcomes have been reported for patients with CRC and *KRAS* mutations than for those with wild-type *KRAS* CRC (26, 28). Bevacizumab combined with chemotherapy is the recommended first-line treatment for patients with mCRC (29) as it prolongs PFS by 2–6 months, irrespective of *KRAS* mutation status (30, 31). However, limited data are available on the impact of *KRAS* mutation status on clinical outcomes in patients with mCRC treated with other anti-angiogenic agents, such as the bispecific antibody vanucizumab, which targets both VEGF-A and Ang-2.

There are currently no known predictors for the outcome of anti-angiogenic treatment. Different trials have shown mixed data on some biomarkers (e.g. VEGF-A, endothelial nitric oxide synthase, VEGFR1/R2, *KRAS* mutation status) (24, 32–35), but no clear predictors have been identified in patients with mCRC receiving anti-angiogenic/anti-VEGF treatment. The aim of this exploratory analysis was to investigate the predictive potential of biomarkers, including Ang-2, in patients with mCRC treated with vanucizumab or bevacizumab, both plus mFOLFOX-6, in the McCAVE study. We examined biomarker levels in tumour tissue and plasma (Ang-2 only) samples, and given the importance of *KRAS* mutations on survival in patients with mCRC, we stratified patients by *KRAS* mutation status.

## 2 Methods

In the phase II McCAVE study (NCT02141295), previously untreated patients with mCRC were randomised to receive either vanucizumab/mFOLFOX-6 or bevacizumab/mFOLFOX-6. The study design, patient characteristics and treatment details have been published (23). All patients provided written informed consent as approved by local institutional review boards.

### 2.1 Tissue biomarker sampling and analysis

Tumour tissue (from surgical specimens or biopsies) were collected from all participating patients before treatment and analysed separately by treatment arm.

Archival tumour tissue samples were obtained, embedded in paraffin blocks and sectioned (HistogeneX, now CellCarta, Antwerp, Belgium). Eight 2.5–4.0 µm thick sections per tumour block were processed according to routine histology and immunohistochemistry (IHC) protocols. Sections were stained with haematoxylin and eosin (H&E) or subjected to chromogenic brightfield simplex, duplex or triplex assays developed and validated at the Roche Innovation Center Munich (Penzberg, Germany). Tumour samples were assessed histologically and only those determined to be from a primary CRC were included in the biomarker and mutational status exploratory analysis presented here.

Details on IHC assays used for staining for the various biomarkers analysed in tumour tissue samples are given in **Table 1**. Ang-2 (biomarker of angiogenesis) and CD34 (biomarker of vessels in the total tumour vasculature) were assessed using duplex staining for Ang-2 (ANGPT2)/CD34. Perforin (PRF1, cytolytic protein expressed by CD8+ T-cells)/CD3 (total T-cell marker), MKi67 (proliferating T-cell marker)/CD8 (cytotoxic T-cell marker) and Forkhead box P3 (FOXP3, regulatory T-cell marker) staining was used to assess densities of lymphocyte subpopulations. CD163+ CD68+ staining was used to assess the percentage of area coverage of M2 macrophages in the tumour area, the cleaved form of caspase 3 (CLEAVED CASP3 [CC3]) was used as a marker for apoptosis and carbonic anhydrase isoform 9 (CA9) was used as a marker of hypoxia.

**Table 1:** Staining details for biomarkers analysed in tumour tissue samples.

Biomarker	Antibody clone	Detection system	Staining instrument
Ang-2 (ANGPT2)/CD34 duplex staining	Ang2 clone K-20H6 (Roche Diagnostics GmbH) (monoclonal rabbit Ab) self-prepared dispenser CD34 clone QBEnd/10 (Ventana Medical Systems) (monoclonal mouse Ab) ready to use dispenser	Ultraview AP Red (CD34) (Ventana 760-501) Optiview DAB (Ang-2) (Ventana 760-099)	Ventana Discovery XT
Perforin/CD3 duplex staining	Anti-PRF1 clone 5B10 (Abcam) (monoclonal mouse Ab) self-prepared dispenser Anti-CD3E clone 2GV6 (Ventana Medical Systems) (monoclonal rabbit Ab) ready to use dispenser	Ultraview AP Red (CD3) (Ventana 760-501) Optiview DAB (perforin) (Ventana 760-099)	Ventana Discovery Ultra
MKi67/CD8 duplex staining	MKi67 clone 30-9 (Ventana Medical Systems) (monoclonal rabbit Ab) ready to use dispenser CD8 clone SP239 (Spring bioscience) (monoclonal rabbit Ab) self-prepared dispenser	Ultraview AP Red (CD8) (Ventana 760-501) Optiview DAB (MKi68) (Ventana 760-099)	Ventana Discovery Ultra
FOXP3	236A/E7 (CNIO, Madrid) (monoclonal mouse Ab) self-prepared dispenser	Optiview DAB (Ventana 760-099)	Ventana Benchmark XT
CD163/CD68 duplex staining	Anti-CD163 clone MRQ-26 (Cell Marque) (monoclonal mouse Ab) ready to use dispenser Anti-CD68 clone PG-M1 (DAKO) (monoclonal mouse Ab) ready to use dispenser	Ultraview AP Red (CD68) (Ventana 760-501) Optiview DAB (CD163) (Ventana 760-099)	Ventana Discovery XT
CC3/CA9/MKi67 triplex staining	CASP3 clone J20H1L1 (Spring Bioscience) (monoclonal rabbit Ab) self-prepared dispenser CA9 clone 1G7 (Origene Technologies) (monoclonal mouse Ab) self-prepared dispenser MKi67 clone 30-9 (Ventana Medical Systems) (monoclonal rabbit Ab) ready to use dispenser	Iview Blue (CC3) (Ventana 760-097) Optiview DAB (CA9) (Ventana 760-700) Ultraview Red (MKi67) (Ventana 760 501)	Benchmark XT

Ab, antibody.

All chromogenic simplex, duplex or triplex assays are brightfield and were developed and validated at the Roche Innovation Center Munich.

### 2.2 Automated tissue image analyses and visual slide assessments

Tissue slides were scanned at 20× magnification using a high-throughput whole-slide scanner (Ventana iScan HT, Ventana Medical Systems, Inc., Tucson, AZ, USA). Tissue sample quality and consistency of staining were assessed at the Roche Innovation Center Munich, Germany. A digital pathology algorithm was used to detect the tissue area on the slide. Tumour, necrotic and exclusion areas were annotated manually by a certified pathologist according to internal guidelines. Digital whole-slide scans were subjected to automated image analysis using the in-house developed IRIS digital pathology platform, where the images were scored using dedicated whole-slide automated image analysis algorithms written in Matlab (www.mathworks.com).

The digital pathology algorithms included a colour deconvolution step for stain unmixing (36), followed by a candidate extraction step. Machine learning classification (random forest, logistic regression with L1 regularisation or support vector machine) was used to classify different phenotypes based on a set of features and to remove non-specific stained structures. The *x* and *y* coordinates of the detected objects were recorded and displayed in the IRIS viewer in the form of polygons or seeds (cell centroid).

Algorithm results overlayed on the tissue images were visually checked for accuracy by a pathologist who also manually annotated and excluded image artefacts. Tissue annotations and algorithm results (*x,y* coordinates and respective labels) were stored in a spatial database for further data analysis.

The reports generated were cell densities (number of cells per mm^2^) for the phenotypes total CD3+, PRF1+ CD3E+, PRF1+ CD3E-, total CD8+, MKi67+ CD8A+, MKi67- CD8A+, FOXP3+, MKi67+, CC3+, and CA9+, vessel densities (number of vessels per mm^2^) for the phenotypes ANGPT2+ CD34+ (Ang-2) and CD34+ (total), and ratios for (ANGPT2+ CD34+)/CD34+ (relative amount of Ang-2+ vessels to total number of vessels), (PRF1+ CD3E+)/total CD3+ (relative amount of natural killer T cells to total CD3), (MKi67+ CD8A+)/total CD8+ (relative amount of proliferating CD8 to total CD8) and CD163+ CD68+ (percentage of area coverage of M2 macrophages in tumour area).

All digital pathology scoring algorithms were verified for performance during a development phase before use on clinical trial data. Detailed descriptions can be found in the **Supplementary Material**.

### 2.3 Plasma sampling and analysis

Blood (approximately 6 mL) samples were taken prior to the receipt of treatment for the determination of free and total Ang-2 circulating levels. Samples were stabilised in K3-EDTA. Free Ang-2 levels were assessed using an enzyme-linked immunosorbent assay (Quantikine^®^). Analytical methods have been reported in more detail (21, 37).

### 2.4 Determination of KRAS mutation status

Specimens with >50% tumour content were macro-dissected from archival formalin-fixed paraffin-embedded tumour samples, and the DNA was extracted. Only samples meeting the minimum amplifiable DNA copy number for sequence enrichment (quantified using Asuragen’s QuantideX^®^ DNA QC assay) (38) were processed further. Sequence enrichment and library preparation were carried out using the QuantideX^®^ Pan Cancer kit, followed by next generation sequencing (NGS) (Illumina MiSeq^®^ system) (39). Target median amplicon coverage was 1000-fold. The QuantideX^®^ NGS Pan Cancer panel interrogates 46 gene regions (amplicons) within 21 oncogenes, including *KRAS* (codon regions 4–15, 55–65, 104–118 and 137–148; for a full list of oncogenes see Kelnar et al. (40)). Patients were classified as having mutated or wild-type *KRAS*.

### 2.5 Statistical analysis

All patients randomised to treatment with either vanucizumab/mFOLFOX-6 or bevacizumab/mFOLFOX-6 for whom data on *KRAS* mutation status were available were included in this exploratory analysis. To assess the predictive potential of biomarkers, the association between PFS and the density of various biomarkers in tissue samples and circulating levels of free Ang-2 in patient plasma samples was explored. PFS was the primary endpoint of the McCAVE clinical trial and was defined as the time from randomisation to the date of first documented occurrence of progression based on Response Evaluation Criteria In Solid Tumors (RECIST) version 1.1 criteria) (41), as determined by the investigator, or death from any cause on study, whichever occurred first.

All analyses were performed separately in tissue and plasma samples from each of the two study arms, stratified by *KRAS* mutation status (wild-type vs mutated). Biomarker density in tumour samples and baseline Ang-2 levels in plasma were classified as higher than (high) or lower/equal (low) to the median value. Median PFS for each treatment group by biomarker level (high or low) and by *KRAS* mutation status (wild-type or mutated) was estimated using Kaplan–Meier plots. Between-group differences in PFS were compared statistically using univariate Cox models. For each of the specified subgroups, hazard ratios (HRs) and 95% confidence intervals (CIs) were calculated for the vanucizumab/mFOLFOX-6 arm relative to the bevacizumab/mFOLFOX-6 arm using Cox regression. Statistical analyses were conducted using JMP^®^, Version 15.2.0. SAS Institute Inc., Cary, NC, USA, 1989–2021.

## 3 Results

### 3.1 Patient population

**Figure 1:**
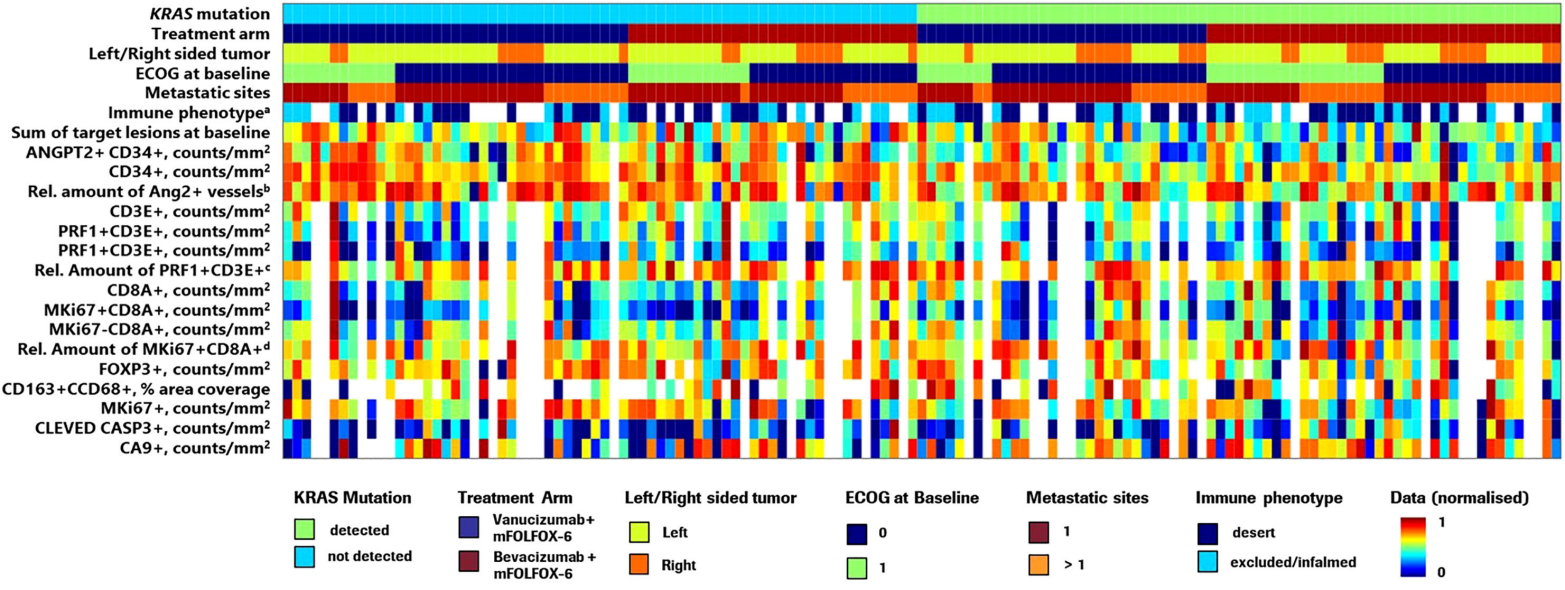
Heatmap showing the distribution of McCAVE study patients by KRAS mutation status and treatment arm providing an overview of per patient clinical information and tissue biomarker densities. ECOG, Eastern Cooperative Oncology Group; mFOLFOX, modified folinic acid (leucovorin), 5-fluorouracil and oxaliplatin; Rel., relative. Values were normalised in the range 0–1. ^a^Immunophenotyping (pre-existing tumour immune contexture) analysis was performed on MKi67/CD8-stained slides using an adaptation of density proportion score methodology (40). ^b^(ANGPT2+ CD34+)/CD34+ ratio – relative amount of Ang-2+ vessels to CD34+ (total number of vessels). ^c^(PRF1+ CD3E+)/total CD3+ ratio – relative amount of natural killer T cells to total CD3. ^d^(MKi67+ CD8A+)/total CD8+ – relative amount of proliferating CD8 to total CD8. Note: Due to lack of information on some demographic variables for 2 patients, these patients were not included in this visualisation.

Of 189 patients enrolled in the McCAVE phase II study, 94 were randomised to the vanucizumab/mFOLFOX-6 arm and 95 were randomised to the bevacizumab/mFOLFOX-6 arm. Baseline median age (64.0/63.0 years), and proportion of patients with left-sided tumours (75.3%/61.1%) or >1 metastatic site (63.8%/63.2%) were broadly comparable for the two groups; however, greater proportions of participants receiving vanucizumab/mFOLFOX-6 were male (59.6%/40.0%) and had an ECOG performance 0 (63.8%/49.5%) **(Supplementary Table 1**). *KRAS* mutation status data were available for 80 patients receiving vanucizumab/mFOLFOX-6 and 81 receiving bevacizumab/mFOLFOX-6; 37 (46.3%) and 45 (55.6%), respectively, carried a *KRAS* mutation. A breakdown of the *KRAS* mutation landscape (**Supplementary Figure 1**) shows that the *KRAS* mutations primarily occurred at codons 12 and 13 of exon 2. A heatmap of the McCAVE study cohort at baseline by known *KRAS* mutation status and treatment arm is presented in **Figure 1**. This provides a descriptive overview of baseline patient population information and associated per-patient tissue biomarker densities. There is no clear relationship between Ang-2 expression and the listed patient demographics.

### 3.2 Biomarker analyses

**Figure 2:**
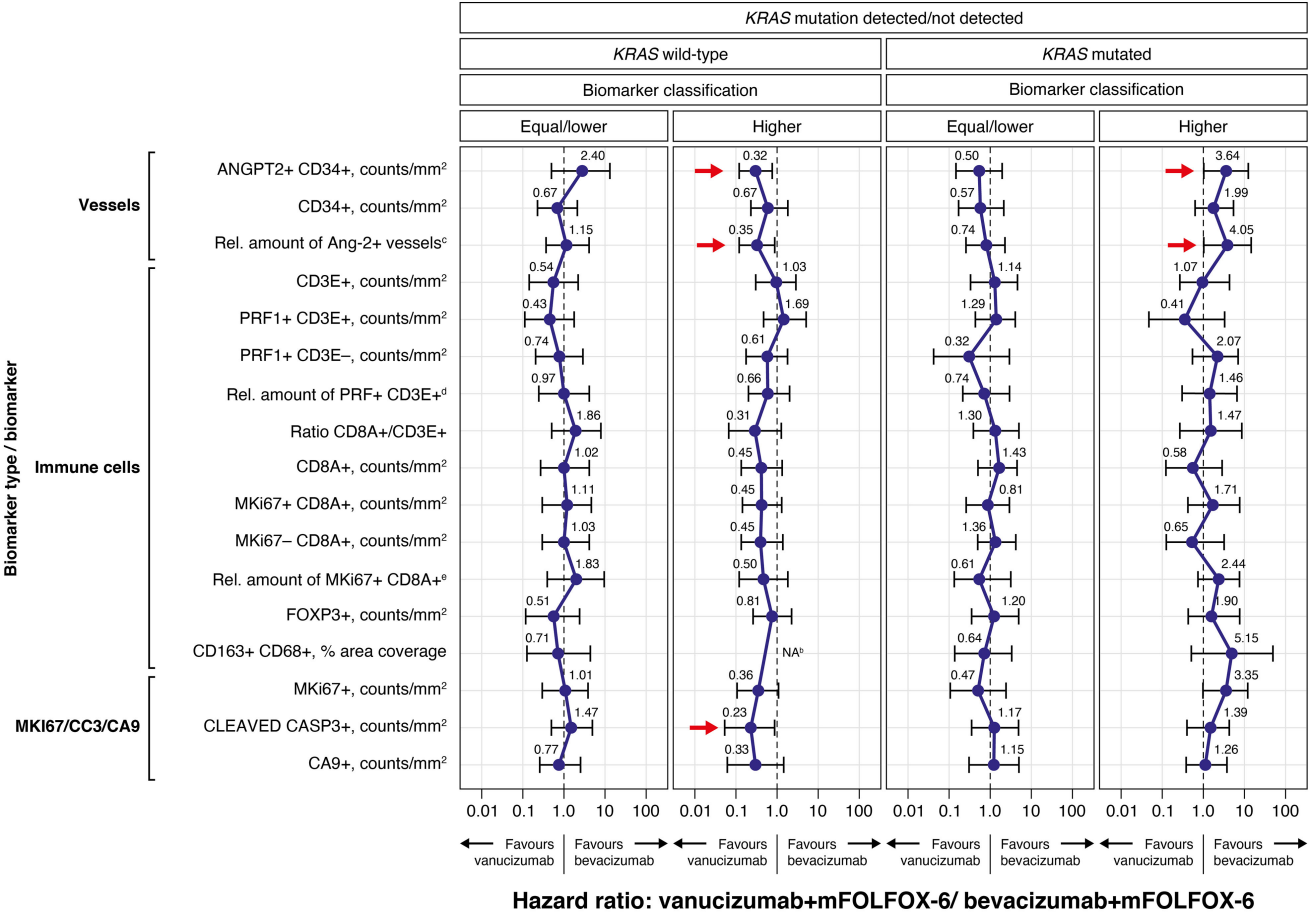
Forest plots of progression-free survival (PFS) hazard ratios (95% CI) for the vanucizumab/mFOLFOX-6 arm relative to the bevacizumab/mFOLFOX-6 arm for each tissue biomarker, dichotomised by the median value^a^, and by *KRAS* mutation status (wild-type vs mutated), calculated using Cox regression. Each error bar is constructed using the minimum and maximum of the data. mFOLFOX, modified folinic acid (leucovorin), 5-fluorouracil and oxaliplatin; Rel., relative. ^a^Median values for each biomarker can be found in **Supplementary Table 2**. ^b^The hazard ratio estimation for CD163+ CD68+macrophages not shown as the upper confidence interval could not be reliably estimated (however, the point estimate was close to zero). ^c^(ANGPT2+ CD34+)/CD34+ ratio – relative amount of Ang-2+ vessels to CD34+ (total number of vessels). ^d^(PRF1+ CD3E+)/total CD3+ – relative amount of natural killer T cells to total CD3. ^e^(MKi67+ CD8A+)/total CD8+ – relative amount of proliferating CD8 to total CD8.

**Figure 2** presents the Forest plots of PFS HRs (95% CI) of vanucizumab/FOLFOX-6 versus bevacizumab/FOLFOX-6 stratified for each tissue biomarker dichotomised by its median value (see **Supplementary Table 2**) and by *KRAS* mutations status. In patients with wild-type *KRAS* and high baseline densities of Ang-2+ vessels (ANGPT2+ CD34+), there was a PFS benefit with vanucizumab-based treatment over bevacizumab/mFOLFOX-6, as demonstrated by the 95% CIs of the HR below 1. A similar finding was observed for the subgroup of *KRAS* wild-type patients with a high relative amount of Ang-2+ vessels to the total number of vessels.

An analogous observation (95% CIs of the HR below 1) was also seen in patients with wild-type *KRAS* and high levels of CC3, again indicating a PFS benefit with vanucizumab/FOLFOX-6 over bevacizumab/mFOLFOX-6. Higher than median baseline levels of MKi67 and CA9 showed a trend towards a PFS benefit (upper 95% CI of the HR just over 1) in patients with wild-type *KRAS* treated with vanucizumab/mFOLFOX-6 versus bevacizumab/mFOLFOX-6.

In patients with mutant *KRAS* and high baseline densities of Ang-2, the PFS benefit favoured bevacizumab/FOLFOX-6 (95% CIs of the HRs above 1). In this sub-population, the relative amount of Ang-2+ vessels also correlated with a favourable clinical outcome. Likewise, high median baseline levels of MKi67 showed a trend towards a PFS benefit (lower 95% CI of the HR just below 1) in patients with mutant *KRAS* treated with bevacizumab/mFOLFOX-6 versus vanucizumab/mFOLFOX-6.

As a clear PFS benefit (i.e. 95% CIs of the HRs above or below 1) for either bevacizumab/mFOLFOX-6 or vanucizumab/mFOLFOX-6 was observed for higher than median baseline levels of Ang-2, and as the additional blockade of this angiopoietin represents the main difference in mode of action between bevacizumab and vanucizumab, we decided to focus on the Ang-2 analysis in more detail.

### 3.3 Ang-2 tissue analysis

Data on *KRAS* mutation status and Ang-2 in tissue samples were available for 139 patients (68 receiving vanucizumab/mFOLFOX-6 and 71 bevacizumab/mFOLFOX-6) (**Table 2A**); 71 (51%) of whom had mutant *KRAS*.

**Table 2:** Median progression-free survival, estimated using Kaplan-Meier methodology, stratified by *KRAS* mutation status and treatment arm.

(A) Tissue samples										
*KRAS wild-type (n=68)*										
		Vanucizumab/mFOLFOX-6 (n=38)			Bevacizumab/mFOLFOX-6 (n=30)			Difference in PFS** (days)	HR (95% CI)^a^	Log-rank p-value
		n	PFS (days)	Lower–upper 95% (days)	n	PFS (days)	Lower–upper 95% (days)			
Ang-2+	Higher***	24	386	320–559	17	223	170–338	163	0.32 (0.13; 0.82)	p=0.01^b^
	Lower***	14	304	304–337	13	445	200–NA	-141	2.40 (0.46; 12.46)	p=0.17
*KRAS mutation (n=71)*										
		Vanucizumab/mFOLFOX-6 (n=30)			Bevacizumab/mFOLFOX-6 (n=41)			Difference in PFS** (days)	HR (95% CI)^a^	Log-rank p-value
		n	PFS (days)	Lower–upper 95% (days)	n	PFS (days)	Lower–upper 95% (days)			
Ang-2+	Higher***	12	219	56–343	17	394	225–459	-175	3.64 (1.05; 12.60)	p=0.01^b^
	Lower***	18	381	237–NA	24	309	222–515	72	0.50 (0.14; 1.80)	p=0.16
(B) Plasma samples										
*KRAS wild-type (n=78)*										
		Vanucizumab/mFOLFOX-6 (n=42)			Bevacizumab/mFOLFOX-6 (n=36)			Difference in PFS** (days)	HR (95% CI)^a^	Log-rank p-value
		n	PFS (days)	Lower–upper 95% (days)	n	PFS (days)	Lower–upper 95% (days)			
Ang-2	Higher***	21	361	304–386	17	224	200–282	137	0.39 (0.14; 1.02)	p=0.048^b^
	Lower***	21	394	337–NA	19	486	284–NA	-92	0.84 (0.26; 3.03)	p=0.83
*KRAS mutation (n=78)*										
		Vanucizumab/mFOLFOX-6 (n=35)			Bevacizumab/mFOLFOX-6 (n=43)			Difference in PFS** (days)	HR (95% CI)^a^	Log-rank p-value
		n	PFS (days)	Lower–upper 95% (days)	n	PFS (days)	Lower–upper 95% (days)			
Ang-2	Higher***	16	343	62–NA	17	292	175–459	51	0.97 (0.32; 2.96)	p=0.96
	Lower***	19	265	219–NA	26	338	222–444	-73	0.99 (0.33; 2.96)	p=0.99

(A) By baseline Ang-2 densities in tissue samples (n=139)*; (B) by baseline plasma angiopoietin-2 concentration (n=156)*. *Patients for whom sufficient tumour tissue was available or for whom DNA extraction was successful.

**PFS (vanucizumab/mFOLFOX-6) – PFS (bevacizumab/mFOLFOX-6).

***Baseline Ang-2+ densities/Ang-2 levels were classed as higher or lower than the median value: 85.2 and 22.0 counts/mm^2^ in biopsies and surgical specimens, respectively/3.0 ng/mL in plasma samples.

a HRs and 95% CIs calculated using univariate Cox regression.

b Value is significant.

Ang-2, angiopoietin-2; CI, confidence interval; HR, hazard ratio; NA, not available; PFS, progression-free survival.

**Figure 3:**
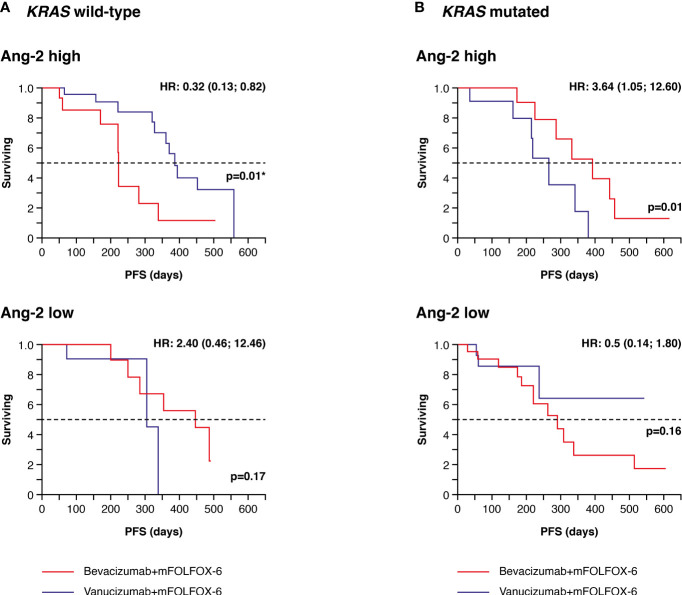
Kaplan–Meier plots of progression-free survival (PFS) in tumour tissue samples. **(A)** Patients with wild-type *KRAS* and higher (high) or lower/equal (low) median levels of baseline angiopoietin-2 (Ang-2)^a^ treated with vanucizumab/mFOLFOX-6 or bevacizumab/mFOLFOX-6. **(B)** Patients with mutant *KRAS* and high or low baseline angiopoietin-2 (Ang-2) treated with vanucizumab/mFOLFOX-6 or bevacizumab/mFOLFOX-6. Numbers of patients at risk at each time point are shown in **Supplementary Table 3**. mFOLFOX, modified folinic acid (leucovorin), 5-fluorouracil and oxaliplatin. ^a^Median values for Ang-2 can be found in **Supplementary Table 2**. Hazard ratios (HRs) and 95 confidence intervals (CIs) were calculated using univariate Cox regression. p-values are from the log-rank test. ^*^Value is significant.

High densities of Ang-2+ vessels were associated with a significantly longer PFS in patients with wild-type *KRAS* treated with vanucizumab when compared with those who received bevacizumab (median 386 vs 223 days, difference: 163 days in favour of vanucizumab, p=0.01; see Kaplan–Meier curves **Figure 3A** and **Table 2A**). This trend was not seen in *KRAS* wild-type patients with low Ang-2+ vessel densities or in *KRAS* mutant patients with high or low Ang-2+ vessel densities (**Figure 3B**). Indeed, in KRAS mutant patients, high densities of Ang-2+ vessels were associated with a significantly longer PFS in patients treated with bevacizumab when compared with those who received vanucizumab (median 394 vs 219 days, difference: 175 days in favour of bevacizumab, p=0.01; **Table 2A**).

**Figure 4:**
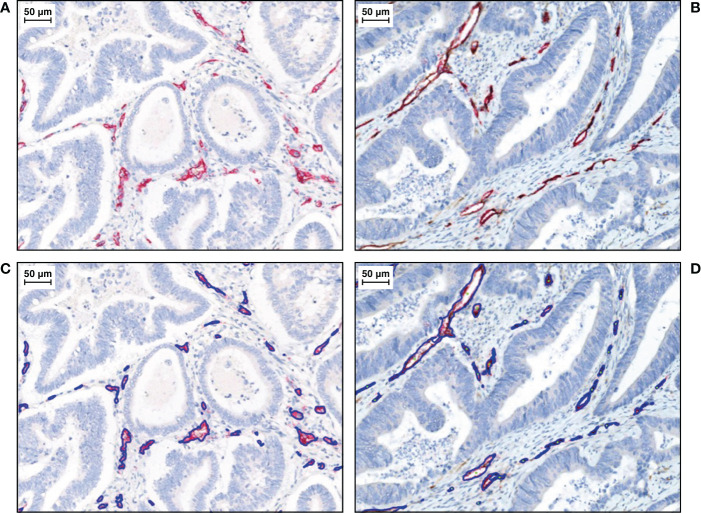
Representative IHC images of duplex Ang-2+ CD34+ tissue staining, with CD34+ endothelial cells stained in red (total vessel population) and Ang-2+ endothelial cells stained in DAB (brown). Haematoxylin is stained in blue. **(A)** Lower than median and **(B)** higher than median.^a^With algorithm results overlays on **(C)** lower than median and **(D)** higher than median. IHC, immunohistochemistry.

Representative IHC images of higher than median and lower than median Ang-2+ CD34+ tissue staining are shown in **Figure 4**.

Information on response to treatment was available for 134 patients (64 receiving vanucizumab/mFOLFOX-6 and 70 bevacizumab/mFOLFOX-6). Best overall response according to median tissue density of Ang-2+ and stratified by *KRAS* mutation status is shown in **Table 3**.

**Table 3:** Best overall response according to median Ang-2+ density.

*KRAS* mutation status	Treatment arm	Ang-2+ median density	Best overall response*			
			Progressive disease	Stable disease	Partial response	Complete response
KRAS wild-type	Vanucizumab/mFOLFOX-6	Higher**	0	7	17	0
		Lower**	0	8	6	0
	Bevacizumab/mFOLFOX-6	Higher**	2	5	8	1
		Lower**	0	3	9	1
KRAS mutation	Vanucizumab/mFOLFOX-6	Higher**	2	9	1	0
		Lower**	1	5	7	1
	Bevacizumab/mFOLFOX-6	Higher**	0	8	10	0
		Lower**	2	11	10	0

*Assessed according to Response Evaluation Criteria in Solid Tumours (RECIST) 1.1 criteria.

**Baseline Ang-2+ densities/Ang-2 levels were classed as higher or lower than the median value: 85.2 and 22.0 counts/mm^2^ in biopsies and surgical specimens, respectively/3.0 ng/mL in plasma samples.

### 3.4 Ang-2 plasma analysis

To confirm the results obtained in tissue samples, we also investigated the association between higher and lower than median plasma levels of Ang-2 and PFS in patients with and without mutant *KRAS* tumours.

**Figure 5:**
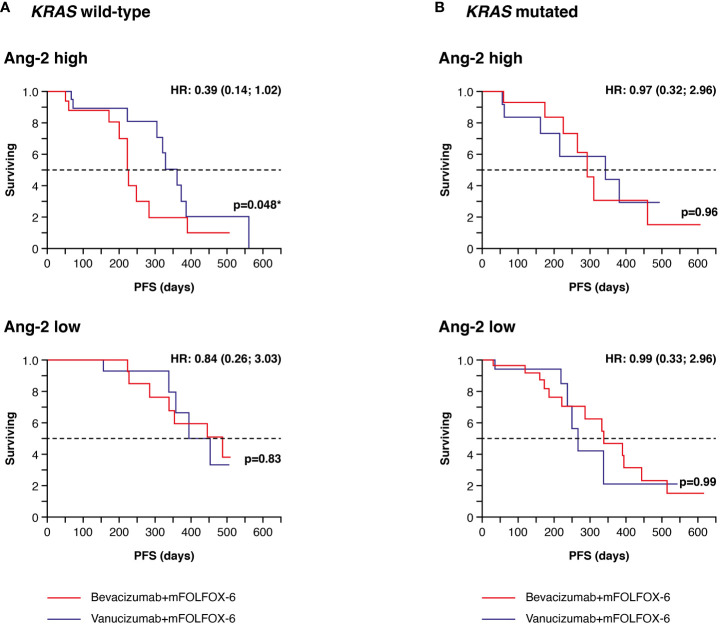
Kaplan–Meier plots of progression-free survival (PFS) in plasma samples. **(A)** Patients with wild-type *KRAS* and higher (high) or lower/equal (low) median levels of baseline angiopoietin-2 (Ang-2)^a^ treated with vanucizumab/mFOLFOX-6 or bevacizumab/mFOLFOX-6. **(B)** Patients with mutant *KRAS* and high or low baseline Ang-2 treated with vanucizumab/mFOLFOX-6 or bevacizumab/mFOLFOX-6. Numbers of patients at risk at each time point are shown in **Supplementary Table 3**. mFOLFOX, modified folinic acid (leucovorin), 5-fluorouracil and oxaliplatin. ^a^Median values for Ang-2 can be found in **Supplementary Table 2**. Hazard ratios (HRs) and 95 confidence intervals (CIs) were calculated using univariate Cox regression. p-values are from the log-rank test. ^*^Value is significant.

Data on *KRAS* mutation status and Ang-2 in plasma were available for 156 patients (77 receiving vanucizumab/mFOLFOX-6 and 79 receiving bevacizumab/mFOLFOX-6); 78 (50%) of whom had mutant *KRAS* tumours (**Table 2B**). In patients with wild-type *KRAS* and higher than median baseline Ang-2 levels (see **Supplementary Table 2**), median PFS estimated from the Kaplan–Meier curves was significantly longer (median PFS 361 vs 224 days; difference: 137 days, p=0.048) in patients who received vanucizumab/mFOLFOX-6 than in those treated with bevacizumab/mFOLFOX-6 (**Figure 5A**; **Table 2B**).

No similar trend was seen in *KRAS* wild-type patients with low Ang-2 levels or in *KRAS* mutant patients with high or low Ang-2 levels (**Figure 5B**). The above-reported benefit of bevacizumab in *KRAS* mutant patients with high tissue densities of Ang-2+ was not confirmed in plasma sample analyses.

## 4 Discussion

The aim of this exploratory analysis, conducted in patients with mCRC, was to identify potential predictive biomarkers for a survival benefit with anti-angiogenic treatment. Given its reported impact on patient survival (26, 28), patients were stratified by *KRAS* mutation status. Identifying predictors for the outcome of anti-angiogenic treatment in patients with mCRC could eventually guide the development of patient enrichment strategies.

In this study, in mCRC patients with wild-type *KRAS*, higher than median tissue baseline densities of Ang-2 positive vessels were associated with a significant PFS benefit of 163 days (~5.5 months) in patients treated with vanucizumab/mFOLFOX-6 versus those treated with bevacizumab/mFOLFOX-6. Similar findings were seen in plasma samples from wild-type *KRAS* patients, with high baseline Ang-2 levels associated with a PFS benefit of 137 days in those treated with vanucizumab/mFOLFOX-6 versus bevacizumab/mFOLFOX-6.

Previous research has suggested that Ang-2 is a useful prognostic factor in mCRC patients, with high baseline levels associated with shorter overall survival in a number of studies (e.g. Jary et al. (15), Goede et al. (19), Chung et al. (42)). Previously reported results from the McCAVE study found that baseline plasma Ang-2 levels were prognostic for PFS in patients receiving vanucizumab or bevacizumab plus chemotherapy; high Ang-2 plasma levels at baseline were associated with a shorter PFS compared with low levels (23).

Consistent with these findings, the current exploratory analysis shows that, overall, low baseline levels of Ang-2 were associated with longer PFS than high Ang-2 levels, especially in patients with wild-type *KRAS* status. Although wild-type *KRAS* is generally associated with a better prognosis than mutated *KRAS* in patients with CRC (26, 28, 43), the results of our study demonstrate additionally, for the first time, that those wild-type *KRAS* patients who were at risk of a poorer outcome (i.e. those with high Ang-2 levels) had a significant PFS benefit if they received vanucizumab treatment instead of bevacizumab. The likely mechanism underlying this observation is that the additional blocking of Ang-2 signalling pathways with vanucizumab counteracts the Ang-2 upregulation escape mechanism that has previously been described (11). None of the above-mentioned studies reporting on the prognostic significance of Ang-2 in mCRC examined patients by *KRAS* mutation status (15, 19, 23, 42). A study by Peeters et al. (44), which found no association between baseline Ang-2 levels and PFS in patients with mCRC receiving trebananib, an investigational peptide-Fc fusion protein that neutralises the interaction between angiopoietins-1/-2 and the Tie2 receptor, with or without chemotherapy, did examine patients by *KRAS* mutation status, but found no evidence that this impacted results. However, Peeters et al. (44) did not report any subgroup analysis of biomarkers (e.g. Ang-2) according to *KRAS* mutation status.

The observed significant PFS benefit for high density of Ang-2 positive vessels in *KRAS* wild-type patients treated with vanucizumab was accompanied by a parallel result for high levels of CC3. This biomarker of apoptosis showed a PFS benefit for vanucizumab over bevacizumab when present at baseline in higher than median levels in the *KRAS* wild-type subpopulation (**Figure 2**). Also of note is a similar trend shown by CA9 (hypoxia) and MKi67 (proliferation), which could be indicative that the fast growth of the tumour in these previously untreated patients is not being supported at the same rate by the formation of new tumour neo-vascularisation, resulting in apoptosis triggered by hypoxia (45), and for CD8-related phenotypes.

Altogether our data highlight the interplay between these biomarkers in the underlying tumour growth mechanism. The increased need for oxygen and nutrients by growing tumours, added to the immature and inefficient tumour-associated vasculature, leads to a hypoxic microenvironment (46) that activates the Ang-2 signalling pathway, providing further vessel sprouting and, hence, potentiating angiogenesis (47, 48). Indeed, Ang-2 has been shown to be present in higher concentrations only at sites undergoing vascular remodelling and in a hypoxic tumour microenvironment (48). With our data showing that *KRAS* wild-type patients with high densities at baseline of Ang-2, CC3 and CA9 benefit from vanucizumab treatment, we hypothesise that in this ‘Ang-2-rich’ group of patients the added inhibition of Ang-2 is more effective in slowing tumour growth and metastasis than VEGF inhibition alone, counteracting tumour escape mechanisms, thus allowing increased levels of vessel normalisation and immune cell infiltration, by upregulation of the expression of adhesion molecules to which T-cells bind in order to cross the endothelial cells layer (49, 50). The normalisation of the tumour vasculature, and more generally of the tumour microenvironment, stimulates T-cell activation (49) and contributes to a more efficient reach of the combined FOLFOX chemotherapy.

We additionally investigated the association of the different patient sub-populations, given by the Ang-2 and KRAS patient stratification, with best overall response (assessed according to Response Evaluation Criteria in Solid Tumours [RECIST] 1.1 criteria) and observed that a greater number of patients responded to vanucizumab than to bevacizumab in the KRAS wild-type high Ang-2 population, which also suggests that this subpopulation benefits more from the dual inhibition of Ang-2 and VEGF-A.

No association was observed between PFS and high/low baseline tissue densities of CD34 (used as a biomarker of vessels), which suggests that the prolongation of PFS observed with vanucizumab versus bevacizumab in patients with wild-type *KRAS* and high levels of Ang-2 is an effect that cannot be extended to the general vessels, and can be considered a result of the additional Ang-2 blockade seen with vanucizumab.

Overall, our current analysis suggests that, although high Ang-2 levels remain a negative prognostic biomarker in mCRC, vanucizumab treatment has the potential to turn this negative prognostic into a positive predictive biomarker in the *KRAS* wild type mCRC subpopulation. It also underscores the importance of investigating biomarker combinations for patient stratification rather than looking at biomarkers, gene mutations, etc., in isolation. In this analysis’s cohort, ~30% of the patients have at baseline both Ang-2 high levels (dichotomised according to the median) and *KRAS* wild-type status (**Table 2**).

In contrast to our above findings, the Forest plot of PFS HRs for vanucizumab/FOLFOX-6 versus bevacizumab/FOLFOX-6 showed that patients with *KRAS* mutations and high tissue Ang-2 positive vessels responded better to bevacizumab than to vanucizumab. This was seen in the analyses of Ang-2 levels in tissue (Ang-2 presence in vessels’ endothelial cells only) but not in plasma. It should be borne in mind that *KRAS* mutations are heterogeneous, and that *KRAS* mutations in different codons dictate a distinct angiogenic profile (51–53), which could impact the efficacy of different administered therapies (51, 52). Hence, targeting Ang-2 may be less effective in a *KRAS*-mutated population. Our *KRAS* mutated mCRC cohort exhibited typical heterogeneity regarding mutation subtypes, with most *KRAS* mutations occurring at codons 12 and 13 of exon 2 (**Supplementary Figure 1**) (53). G12D, the most common subtype identified, has been reported to be significantly associated with poor PFS (43). However, the low patient numbers in each *KRAS* mutation/treatment/Ang-2 subgroup in our dataset precluded further stratification by *KRAS* mutation subtype and, hence, exploration of their association with clinical outcome and potential mechanisms of effect.

Overall, these findings suggest that although Ang-2 status seems to have a potential predictive value in *KRAS*-wild type patients, favouring vanucizumab over bevacizumab, it has no impact on treatment choice and outcome in the *KRAS*-mutated population. Recent advances have been made in the field of KRAS-directed therapy with several registered trials targeting different KRAS mutation variants (54, 55). As our understanding evolves on both angiogenesis and the influence of KRAS, the search for treatment options for these patients with an unmet need for therapies that account for their a priori disadvantage might lead to the investigation of novel combination therapies, similar to previous studies assessing anti-angiogenic and immunotherapy combination treatment (56).

Strengths of our study include that our finding of an association between high levels of Ang-2 and improved PFS in vanucizumab-treated patients with wild-type *KRAS* was seen in tumour tissue data and confirmed in plasma data in separate analyses. The patient subgroups derived from this cohort after stratification were relatively balanced, both in terms of *KRAS* mutation status and tissue biomarker and plasma Ang-2 levels, which precluded the over- or under-representation of specific patient subpopulations. Limitations include that this analysis of McCAVE study data was exploratory, hypothesis-generating, and McCAVE was an early phase clinical trial in which typically the number of enrolled patients is small; hence, the low sample sizes of the patient groups analysed, resulting from the stratification of patients by *KRAS* status and baseline biomarker levels, limit the statistical power of the analysis. An additional limitation is that no post-treatment biomarker or gene mutation status data are available to determine changes over time or in response to treatment. Since this work is an exploratory *post-hoc* analysis, further studies are required for hypothesis confirmation.

Other bispecific antibodies targeting VEGF/Ang-2 have shown promising antitumour activity in preclinical studies and in patients with solid tumours (57, 58). Although the clinical development of vanucizumab for cancer treatment was discontinued following the finding of a similar PFS benefit with vanucizumab and bevacizumab in the overall McCAVE study population (i.e. the primary endpoint of the study was not met), the vessel stabilisation benefit provided by dual inhibition of Ang-2 and VEGF-A was further leveraged in ophthalmology in the treatment of neovascular age-related macular degeneration and visual impairment due to diabetic macular oedema, resulting in the development of the bispecific antibody faricimab (59).

In summary, exploratory analyses of biomarker levels in baseline tumour tissue and plasma samples from patients with previously untreated mCRC stratified for *KRAS* mutation status,suggest a subgroup of patients with *KRAS* wild-type and higher than median levels of baseline Ang-2 in whom vanucizumab/mFOLFOX-6 was associated with a significant survival benefit of ~5.5 months over patients treated with bevacizumab/mFOLFOX-6. Our results indicate that both Ang-2 and *KRAS* mutation status, separately and in combination, are relevant biomarkers in mCRC. This evidence potentially supports the goal of developing more tailored anti-angiogenic treatments for patients with mCRC.

## Data availability statement

The original contributions presented in the analysis are included in the article/**Supplementary Material**. Further inquiries can be directed to the corresponding authors. Details on the commitment of Roche to Data Sharing and the Roche Global Policy on Sharing of Clinical Study Information can be found at https://go.roche.com/data_sharing, where access to the clinical study documents can also be requested.

## Author contributions

CF, GB and FH contributed to conception and design of the presented work. CF, GB, IK, FH, SVH, OK, KL and JB performed data acquisition/processing, data analysis and/or interpretation of data. CF developed dedicated digital pathology software for tissue data analysis and performed data harmonization. GB performed the statistical analysis. CF, GB, IK, FH, SVH, OK, KL and JB contributed to drafting and/or revision of the manuscript. All authors contributed to the article and approved the submitted version.
