# Supplementary material for: Predictive potential of angiopoietin-2 in a mCRC subpopulation treated with vanucizumab in the McCAVE trial
Source: Front Oncol. 2023 May 3;13:1157596. doi: 10.3389/fonc.2023.1157596 (PMC10190963; doi:10.3389/fonc.2023.1157596)

***Supplementary Material***

Predictive Potential of Angiopoietin-2 in a mCRC Subpopulation Treated with Vanucizumab in the McCAVE trial

**Cláudia S Ferreira*, Galina Babitzki*, Irina Klaman, Oliver Krieter, Katharina Lechner, Johanna Bendell, Suzana Vega Harring, Florian Heil**

*** Correspondence:**

**Cláudia S Ferreira** [claudia.ferreira.cf1@roche.com](mailto:claudia.ferreira.cf1@roche.com)

**Galina Babitzki** [galina.babitzki@roche.com](mailto:galina.babitzki@roche.com)

# Supplementary Data

### 1.1 Digital pathology scoring algorithm performance verification

The performance of all scoring algorithms was verified against pathologists’ ground truth (GT) during a development phase before use on clinical trial data. For all the algorithm verifications, the fields of view (FOVs) used were from a separate test set of whole-slide images that was not used for training the algorithm during development.

1.1.1 Ang-2/CD34 algorithm

An image gallery of 200 (FOVs comprising variable numbers and vessel shapes was manually and independently scored by two pathologists who were blind to the algorithm results. GT scoring results were plotted against algorithm findings for the same set of FOVs. The mean positive correlation between the blind scoring method and algorithm results was 90% (Pearson correlation coefficient).

1.1.2 T-cell algorithms

Algorithm-detected seeds (cell centroid, one per cell) were overlaid on a set of 50‒100 FOVs (depending on the abundance in tissue of the cell to be verified) and independently corrected by two pathologists who manually deleted over-detected cells or added missed cells. Original and corrected results were plotted against each other to assess their correlation. T-cell algorithms showed a correlation with the pathologists’ GT of >90%.

1.1.3 Macrophage algorithm

Macrophage area coverage was determined as the area of macrophage staining as a proportion of the area of tumour. The algorithm was verified by comparing the coverage mask derived from a manually (pathologist) set threshold to the mask obtained with an automatically (algorithm) derived threshold. Macrophage detection had a positive correlation with GT of 85%.

1.1.4 MKI67/CC3/CA9 algorithm

The algorithm detection for all markers in the triplex was visually/manually assessed by expert pathologists on a large test image gallery. Algorithm performance was accepted.

## Immunophenotyping

Immunophenotyping (pre-existing tumour immune contexture) analysis was performed on MKi67/CD8-stained slides using an adaptation of density proportion score methodology (Galon, J., Lanzi, A. Immunoscore and its introduction in clinical practice. *Q. J. Nucl. Med. Mol. Imaging.* **64**, 152–161 (2020)). Slides were subjected to visual/manual assessment and the location and abundance of CD8 cells in tumour stroma and epithelial areas was used to score the sample (score provided by HistogeneX, now CellCarta, Antwerp, Belgium) as having one of three immunophenotypes: desert (no to very low infiltration), excluded (infiltrates in the invasive margin but not in the tumour, or infiltrates excluded from the intra-epithelial compartment but abundant in the intra-tumour stroma), or inflamed (infiltration within the intra-epithelial compartment).

**2 Supplementary tables and figures**

**2.1 Supplementary Table 1.** **Baseline demographics of McCAVE study participants (N=189)**

| Patient characteristic | Vanucizumab/mFOLFOX-6 (n=94)* | Bevacizumab/mFOLFOX-6 (n=95) |
| --- | --- | --- |
| Age, years; median (range) | 64 (27-82) | 63 (29-81) |
| Male, n (%) | 56 (59.6) | 38 (40.0) |
| ECOG performance status, n (%)  0  1 | 60 (63.8)  34 (36.2) | 47 (49.5)  48 (50.5) |
| Left/right-sided tumour (exclusive transverse colon), n (%) Left Right | 67 (75.3) 26 (24.7) | 58 (61.1) 37 (38.9) |
| Metastatic sites, n (%) 1 >1 | 34 (36.2) 60 (63.8) | 35 (36.8) 60 (63.2) |
| Adjuvant treatment  Yes  No | 6 (6.4)  88 (93.6) | 7 (7.4)  88 (92.6) |
| *KRAS* mutation status, n (%)  Mutant  Wild-type | n=80  13 (16.3) 67 (83.8) | n=81  11 (13.6)  70 (86.4) |
| *BRAF* mutation status, n (%)  Mutant  Wild-type | n=80  7 (8.8)  73 (91.3) | n=81  5 (6.2)  76 (93.8) |
| Surgery for primary tumour  Biopsy  Surgical sample | 47  32 | 38  40 |

Some data previously reported in Bendell et al. 2020 (20).

^*^One patient randomized to the vanucizumab arm received only chemotherapy before being withdrawn from the study.

Ang-2, angiotensin; *BRAF*, B-rapidly accelerated fibrosarcoma; ECOG, Eastern Cooperative Oncology Group; *KRAS*, Kirsten rat sarcoma virus oncogene.

**2.2 Supplementary Table 2. Median values used to classify baseline tissue and plasma biomarker density as higher than (high) or lower/equal to (low) to the median value**

|  | **Tissue type** | |
| --- | --- | --- |
|  | **Biopsy** | **Surgical specimen** |
| **Biomarker** | **Median** | **Median** |
| ANGPT2+ CD34+, counts/mm^2^ | 85.2 | 22.0 |
| CD34+, counts/mm^2^ | 189.1 | 135.6 |
| Relative amount of Ang2+ vessels^a^ | 44.6 | 15.8 |
| CD3E+, counts/mm^2^ | 274.0 | 195.5 |
| PRF1+ CD3E+, counts/mm^2^ | 72.2 | 69.3 |
| PRF1+ CD3E-, counts/mm^2^ | 8.2 | 5.9 |
| Relative amount of PRF+ CD3E+^b^ | 22.7 | 32.1 |
| CD8A+, counts/mm^2^ | 62.4 | 66.5 |
| MKi67+ CD8A+, counts/mm^2^ | 11.7 | 10.5 |
| MKi67- CD8A+, counts/mm^2^ | 50.7 | 58.0 |
| Relative amount of MKi67+ CD8A+^c^ | 19.4 | 14.7 |
| FOXP3+, counts/mm^2^ | 183.1 | 60.5 |
| CD163+ CD68+, % area coverage | 4.0 | 4.3 |
| MKi67+, counts/mm^2^ | 1748.0 | 544.2 |
| CLEAVED CASP3+, counts/mm^2^ | 92.1 | 81.9 |
| CA9+, counts/mm^2^ | 15.5 | 15.5 |
|  | **Plasma samples** | |
|  | **Median** | |
| Angiopoietin-2, ng/mL | 3.0 | |

^a^(ANGPT2+ CD34+)/CD34+ ratio – relative amount of Ang-2+ vessels to CD34+ (total number of vessels).

^b^(PRF1+ CD3E+)/total CD3+ – relative amount of natural killer T cells to total CD3.

^c^(MKi67+ CD8A+)/total CD8+ – relative amount of proliferating CD8 to total CD8.

**2.3** **Supplementary Table 3. Kaplan–Meier analysis of progression-free survival: numbers of patients at risk at each time point,** **stratified by *KRAS* mutation status and treatment arm. A) By baseline Ang-2 densities in tissue samples; (B) by baseline plasma angiopoietin-2 concentration.**

1. Tissue samples

|  | *KRAS* wild-type | | | | | | | | | | | | | |
| --- | --- | --- | --- | --- | --- | --- | --- | --- | --- | --- | --- | --- | --- | --- |
|  | Ang-2 high* | | | | | | | Ang-2 low* | | | | | | |
| Day | 0 | 100 | 200 | 300 | 400 | 500 | 600 | 0 | 100 | 200 | 300 | 400 | 500 | 600 |
| Vanucizumab/  mFOLFOX-6 | 24 | 23 | 15 | 12 | 6 | 4 | 1 | 14 | 11 | 7 | 4 | 1 | 1 | 1 |
| Bevacizumab/  mFOLFOX-6 | 16 | 11 | 9 | 3 | 2 | 2 | 1 | 15 | 13 | 10 | 7 | 6 | 1 | 1 |
|  | *KRAS* mutation | | | | | | | | | | | | | |
|  | Ang-2 high* | | | | | | | Ang-2 low* | | | | | | |
| Day | 0 | 100 | 200 | 300 | 400 | 500 | 600 | 0 | 100 | 200 | 300 | 400 | 500 | 600 |
| Vanucizumab/  mFOLFOX-6 | 13 | 10 | 6 | 2 | 1 | 1 | 1 | 17 | 13 | 8 | 4 | 3 | 2 | 1 |
| Bevacizumab/  mFOLFOX-6 | 18 | 17 | 10 | 6 | 4 | 2 | 2 | 24 | 19 | 13 | 6 | 4 | 4 | 2 |

1. Plasma samples

|  | *KRAS* wild-type | | | | | | | | | | | | | |
| --- | --- | --- | --- | --- | --- | --- | --- | --- | --- | --- | --- | --- | --- | --- |
|  | Ang-2 high* | | | | | | | Ang-2 low* | | | | | | |
| Day | 0 | 100 | 200 | 300 | 400 | 500 | 600 | 0 | 100 | 200 | 300 | 400 | 500 | 600 |
| Vanucizumab/  mFOLFOX-6 | 19 | 16 | 12 | 9 | 3 | 3 | 1 | 19 | 18 | 10 | 7 | 4 | 2 | 1 |
| Bevacizumab/  mFOLFOX-6 | 15 | 11 | 7 | 3 | 2 | 2 | 1 | 18 | 16 | 14 | 9 | 7 | 2 | 1 |
|  | *KRAS* mutation | | | | | | | | | | | | | |
|  | Ang-2 high* | | | | | | | Ang-2 low* | | | | | | |
| Day | 0 | 100 | 200 | 300 | 400 | 500 | 600 | 0 | 100 | 200 | 300 | 400 | 500 | 600 |
| Vanucizumab/  mFOLFOX-6 | 15 | 10 | 6 | 5 | 3 | 1 | 1 | 17 | 14 | 10 | 3 | 2 | 2 | 1 |
| Bevacizumab/  mFOLFOX-6 | 17 | 14 | 9 | 4 | 3 | 2 | 2 | 23 | 21 | 13 | 8 | 5 | 4 | 2 |

*Baseline Ang-2+ densities/Ang-2 levels were classed as higher or lower than the median value: 85.2 and 22.0 counts/mm^2^ in biopsies and surgical specimens, respectively/3.0 ng/mL in plasma samples.

**2.4 Supplementary Figure 1.** **A breakdown of the *KRAS* mutation landscape in the McCAVE study with *KRAS* mutated metastatic mCRC.**


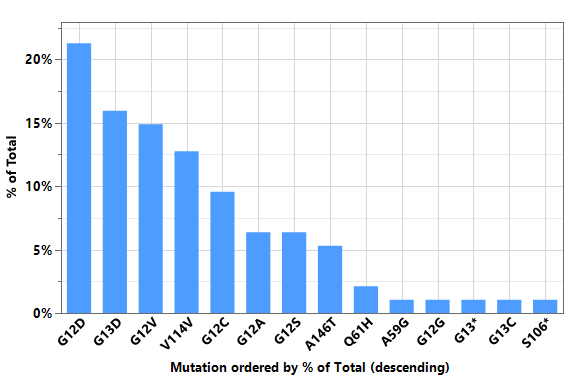

Supplement: Supplementary file 1 [file DataSheet_1.docx]
